# Phenotypic and Transcriptomic Analyses of Transgenic *Arabidopsis thaliana* Expressing Cotton *Zinc Finger Protein8 (GhZFP8)*

**DOI:** 10.3390/genes16091119

**Published:** 2025-09-22

**Authors:** Wenhan Cheng, Chen Rui, Yechuan Huang, Deyan Zhu, Yongchang Liu

**Affiliations:** 1 College of Food and Biology, Jingchu University of Technology, Jingmen 448000, China; chengwenhan@jcut.edu.cn (W.C.);; 2 Hubei Provincial Engineering Research Center for Specialty Flower Bio-Breeding, Jingmen 448000, China

**Keywords:** cotton, zinc finger protein, *GhZFP8*, transcriptome analysis

## Abstract

**Background:** The functional role of *GhZFP8* (a zinc finger protein gene) in plant growth and development remains unclear. **Methods:** This study investigated phenotypic and transcriptomic changes in *GhZFP8*-transgenic vs. wild-type Arabidopsis to clarify *GhZFP8*’s biological functions. Phenotypes of two transgenic lines (ZFP8_7, ZFP8_21) were observed. Transcriptome sequencing (ZFP8_7 vs. wild-type, 3 biological replicates/sample) was performed, with quality assessment (coverage, depth, alignment rate). Differential genes were screened by |log2(fold change)| > 1 and FDR < 0.05; GO/KEGG enrichment analyses were conducted. **Results:** Transgenic lines showed slower growth, higher trichome density, ectopic silique trichomes, and reduced fertility. Sequencing quality was satisfactory (97.42% exonic alignment). Most differential genes were highly expressed in wild-type plants, with more downregulated than upregulated genes. Upregulated genes enriched in stimulus response regulation, plant cell walls, and methyltransferase activity (GO); estrogen signaling/tyrosine metabolism (KEGG). Downregulated genes enriched in cell wall/phenylpropanoid biosynthesis (GO); glycosyltransferase/plant MAPK pathways (KEGG). **Conclusions:** *GhZFP8* overexpression induces significant phenotypic changes and alters gene expression in Arabidopsis. Findings provide insights into *GhZFP8*’s effects, supporting further study of its functional mechanisms.

## 1. Introduction

Cotton, as a fiber-based crop, serves as an indispensable natural raw material for textile production. It generates an annual economic output of approximately US$180–200 billion and supports the livelihoods of 1.8 billion people worldwide engaged in cotton cultivation [1]. In cotton production, critical challenges such as fiber quality and yield optimization, nutrient uptake and utilization under stress conditions, and plant diseases caused by phytopathogens significantly impact crop productivity and quality [2,3,4,5]. To address these challenges, researchers have integrated conventional breeding methods with modern biotechnology, introducing desirable traits from diverse germplasm into target cotton varieties and implementing molecular breeding strategies based on genomics and specific germplasm resources. Genetic modification through transgenic technology represents a fundamental solution for enhancing cotton productivity and addressing environmental constraints [6].

Improvements in cotton fiber yield and quality traits largely depend on elucidating the molecular mechanisms governing fiber development and quality formation, along with the cloning, characterization, and functional studies of key regulatory genes. *Arabidopsis thaliana*, widely recognized as a model plant in plant biology research, including studies on fiber development, is an ideal system for investigating the molecular mechanisms underlying various biological processes, including those relevant to fiber development, which can provide valuable insights into understanding and manipulating similar pathways in cotton. With advancements in molecular biology, functional analysis methods for Arabidopsis genes have become well-established [7]. This study conducted comparative phenotypic analyses between *GhZFP8*-transgenic Arabidopsis thaliana and wild-type plants, focusing on plant height, silique morphology, and growth patterns. Transcriptomic profiling of *GhZFP8*-transgenic Arabidopsis was performed to investigate potential regulatory mechanisms underlying *GhZFP8*-mediated plant growth and development.

Zinc finger proteins (ZFPs) represent a class of transcription factors characterized by finger-like structural domains stabilized by zinc ion coordination. Initially identified in 1983 from Xenopus laevis oocytes through the transcription factor TFIIIA [8], these proteins have subsequently been characterized in various plant species including Arabidopsis thaliana, Oryza sativa, Triticum aestivum, Gossypium hirsutum, and Glycine max, with their functional roles well established. As the largest family of zinc-binding proteins in plants, ZFPs exhibit diverse regulatory functions encompassing trichome formation [9,10,11,12], bud development [13], anther development [14], floral morphogenesis [15], fruit maturation [16], signal transduction, transcriptional regulation, RNA binding, morphogenesis, and stress responses [17,18,19].

Classification of ZFPs into five distinct categories (C2H2, C2C2, C2HC, C2C2C2C2, and C2HCC2C2) is based on conserved cysteine (C) and histidine (H) residues within their zinc-binding domains [20]. Genomic analyses reveal that 29 ZFP genes in upland cotton (Gossypium hirsutum) predominantly originated from whole-genome duplication events, with 20 GhZFP genes arising through this mechanism compared to only two via dispersed duplication. Notably, most *GhZFP8* subfamily members demonstrate high expression during fiber cell elongation, with the exception of *GhZFP8-3* [21]. Cotton fiber development is coordinately regulated by multiple gene families, including TUA, TUB, and MYB transcription factors [22,23,24,25,26,27,28,29]. Although several critical genes have been cloned, the comprehensive regulatory network underlying fiber development remains incompletely elucidated.

GhZFP8, a C2H2-type zinc finger protein localized to both nucleus and cytoplasm, functions as a transcriptional repressor. Expression profiling reveals its specific induction in cotton fibers by phytohormones (auxin, gibberellin, and brassinosteroids) and abiotic stresses (drought, high salinity, low temperature, and Abscisic Acid (ABA) treatment) [30]. The interference of *GhZFP8* in cotton caused smaller bolls and shorter fibers than that of the control. The results of DNA affinity purification (DAP)-seq showed that *GhZFP8* could bind to the promoter, exon, intron, and intergenic region of the target genes, which are involved in photosynthesis, signal transduction, synthesis of biomass [31]. These findings provide a foundation for identifying downstream targets and advancing molecular breeding strategies to enhance cotton fiber traits. Consequently, functional characterization of GhZFP8 represents a critical research priority for elucidating cotton developmental mechanisms and improving crop productivity.

## 2. Materials and Methods

### 2.1. Experimental Materials

Transgenic *Arabidopsis* expressing *GhZFP8* (*GhZFP8*-OE) and wild-type Arabidopsis (Col-0) were used in this study. The transgenic plants used in this study were generated and characterized in our own laboratory, as described in our previously published work [31]. Every fifth plant was harvested as a repeat for mRNA sequencing, and 3 repeats were set for each sample. To investigate the genetic pattern, ntm used as female parent was crossed with WT. Seedings of F1 were self-crossed and offspring of F2 were generated, and then the separation ratio was counted.

### 2.2. Experimental Reagents

The RNA extraction kit was purchased from Tiangen Biotech (Beijing, China). Tris (UltraPure) and acid phenol (pH 4.5) were obtained from Sangon Biotech (Shanghai, China). Sodium lauroyl sarcosinate was a product of Sigma-Aldrich (St. Louis, MO, USA).

### 2.3. Phenotypic Characterization of GhZFP8-Transgenic Arabidopsis Relative to Wild-Type Plants

Transgenic and wild-type Arabidopsis seeds were sterilized and germinated under long-day conditions (16-h light/8-h dark) on 1/2 MS medium. After one week of growth, seedlings of uniform size were transferred to pots containing an equal volume of soil, with five plants per pot and six pots per line. *Arabidopsis thaliana* plants were grown in a mixture of organic nutrient soil and vermiculite (*v*/*v* 3:1) in an incubator. The photoperiod and growth environment were kept consistent, with uniform frequency and volume of watering. No fertilizers were applied throughout the entire growth cycle. Growth phenotypes were photographed and documented at regular intervals. Phenotypic differences between transgenic lines and wild-type plants were systematically observed and compared to investigate the effects of *GhZFP8* overexpression on Arabidopsis growth and development.

### 2.4. RNA Extraction

Total RNA was isolated using the Tiangen RNAsimple Total RNA Extraction Kit (DP419) (Tiangen, Beijing, China). Fresh plant tissue (leaves and stems, 1 g) was ground in liquid nitrogen and processed according to the manufacturer’s protocol. RNA quality was assessed via 1.2% agarose gel electrophoresis after diluting 1 μL of RNA 100-fold. Qualified RNA samples were stored at −80 °C for subsequent analysis.

### 2.5. Transcriptome Analysis of GhZFP8-Transgenic Arabidopsis

A 20 μL reaction mixture containing 15 μL RNA, 4 μL SMART-Frag Buffer, and 1 μL SMART-RT Primer (TSO_PCR 5′-aagcagtggtatcaacgcagagt-3′) was prepared in a 200 μL PCR tube. The mixture was incubated at 85 °C for 3 min in a PCR thermal cycler, followed by immediate cooling on ice. To the reaction mixture, 4.5 μL SMART-RT Buffer, 0.5 μL RNase Inhibitor, and 0.5 μL SMART-RT Enzyme (total volume: 25.5 μL) were added. The mixture was vortexed, incubated at room temperature for 10 min, and then subjected to reverse transcription at 42 °C for 60 min.

A sufficient volume of Stranded Oligo (1 μL per sample) was denatured at 70 °C for 2 min and rapidly chilled on ice. The denatured Stranded Oligo (1 μL) was added to the reverse transcription product, mixed thoroughly, and incubated at 42 °C for 30 min. The reaction product was purified using magnetic beads to remove contaminants.

A 50 μL PCR mixture containing 21 μL purified product, 25 μL PCR Master Mix, 2 μL SMART-Universal Primer, and 2 μL SMART-Primer8 Index N* was prepared. Amplification was performed in a thermal cycler under the conditions specified in Table 1.

### 2.6. Library Purification and Sequencing Preparation

The purified PCR product was adjusted to a final volume of 50 μL using NF-H2O. AMPure XP beads were equilibrated to room temperature and vortex-mixed for 30 min. A volume of 40 μL AMPure XP beads was added to 50 μL PCR product, followed by gentle pipetting (10 cycles) to ensure thorough mixing. The mixture was transferred to a 1.5 mL microcentrifuge tube and incubated at room temperature for 5 min.

### 2.7. Bead Washing and DNA Elution

The tube was briefly centrifuged at low speed and placed on a magnetic rack for 5 min to separate beads from the supernatant. The cleared supernatant was carefully removed. With the tube remaining on the magnetic rack, 200 μL of freshly prepared 80% ethanol was added to wash the beads. After 30 s incubation at room temperature, the ethanol was discarded. A second ethanol wash (200 μL) was performed, followed by a brief low-speed centrifugation and 30 s incubation. Residual ethanol was completely removed. The beads were air-dried on the magnetic rack for 5 min with the tube lid open. The tube was removed from the magnetic rack, and 16 μL NF-H2O was added to elute DNA. The mixture was vortexed thoroughly or gently pipetted to resuspend the beads. After brief centrifugation, the tube was returned to the magnetic rack. Once the solution cleared, 15 μL of supernatant was transferred to a fresh 200 μL PCR tube.

### 2.8. Library Quality Control and Sequencing

The library was qualitatively assessed by agarose gel electrophoresis and quantified using real-time PCR. Libraries meeting quality standards (Typically 10–100 nM for sequencing libraries; ≥5–10 ng/μL for general validation; size 200–2000 bp) were pooled according to experimental requirements. Sequencing was performed on an Illumina NovaSeq 6000 platform with predefined data output parameters.

DESeq2, a standard statistical tool for transcriptome data, were used to perform differential expression analysis of *GhZFP8* between transgenic and wild-type *A. thaliana.* Statistical significance were determined by calculating the adjusted *p*-value (FDR) and log2 fold change (log2FC). Results were presented to clearly demonstrate whether the expression level of *GhZFP8* differs significantly between the two plant types.

### 2.9. Data Processing

Fastp software (0.23.2) was used to filter raw data; remove primers, adaptors, sequences less than 50 bp, reads with a certain proportion of N bases (5 bp by default) and low-quality bases with mass value less than 20; calculate the average mass value of bases with 4 bases. The filtered data was aligned to the rRNA database using Bowtie2 software (2.4.5), and then the rRNA sequence were removed from the sequencing data. Hisat2 (2.2.1) was used to compare the similarity between clean data and the reference genome, obtain the location information of reads on the reference genome and the characterization of samples, and generate BAM files. RseQC software (5.0.1) was used to evaluate the distribution and quality of transcriptome data, such as saturation, RNA degradation and redundant sequence.

### 2.10. GO and KEGG Enrichment Analysis of DEGs

After quality control, the sequences were compared with the reference genome using HISAT2 (2.2.1). Stringtie (2.2.1) was used to detect the expression of known genes, new genes, and transcripts. Differential expression was analyzed using Deseq2 (1.38.2) or edgeR (3.40.2). DEGs require genes or transcripts whose sum of mapping reads ≥ 10 in two samples. The expression fold change was calculated. If |log2 (fold change)| > 1 was met, it was defined as a DEG. The *p* value was corrected by FDR to obtain the Q value, which meets the requirements of *p* value ≤ 0.05 and Q value ≤ 0.05.

GO and KEGG enrichment analysis of DEGs were performed using R package Cluste profiler software (4.6.2). Enrichment analysis was carried out for all DEGs, both up-regulated and down-regulated. According to the results of GO enrichment analysis, DEGs were classified into cellular component (CC), molecular function (MF) and biological process (BP). The 10 GO functions with the most significant enrichment (*p*-value) were selected from the three categories of BP, CC, and MF respectively and displayed in the figure. If there were less than 10 GO functions, they were all listed. In this report, the R software package cluster Profiler (4.6.2) was used for KEGG enrichment analysis.

## 3. Results and Analysis

### 3.1. Phenotypic Analysis of GhZFP8-Transgenic Arabidopsis

**Figure 1:**
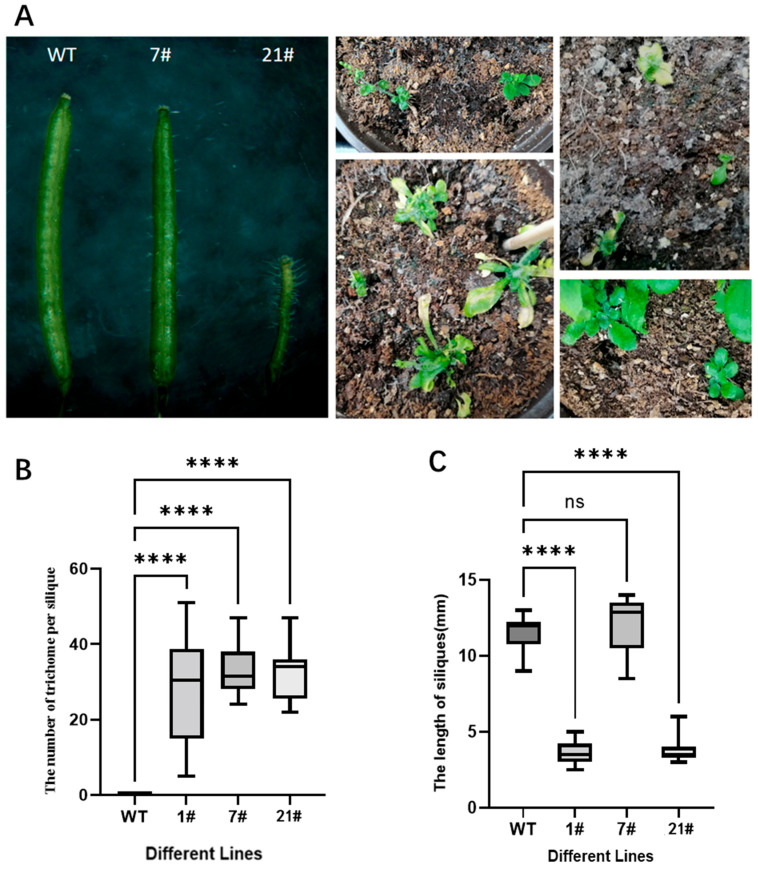
Phenotypes of *GhZFP8* transgenic Arabidopsis thaliana and wild-type *A. thaliana*. ((**A**). Phenotypic photographs of *Arabidopsis thaliana*; (**B**). The number of trichome per silique; (**C**). The length of siliques). **** indicates extremely significant statistical differences and # means number.

Comparative phenotypic observations of *GhZFP8*-transgenic Arabidopsis and wild-type plants under identical growth conditions revealed significant differences. Transgenic lines exhibited markedly slower growth, increased trichome density, and the emergence of ectopic trichomes on siliques. Notably, fertility was reduced in transgenic plants, with severe morphological abnormalities observed in certain lines (Figure 1). Phenotypic quantitative data has already been reported in our previously published article to avoid repetition, and thus is not elaborated here [31].

### 3.2. Genomic Regional Distribution and Overall Transcriptome Quality Assessment

**Figure 2:**
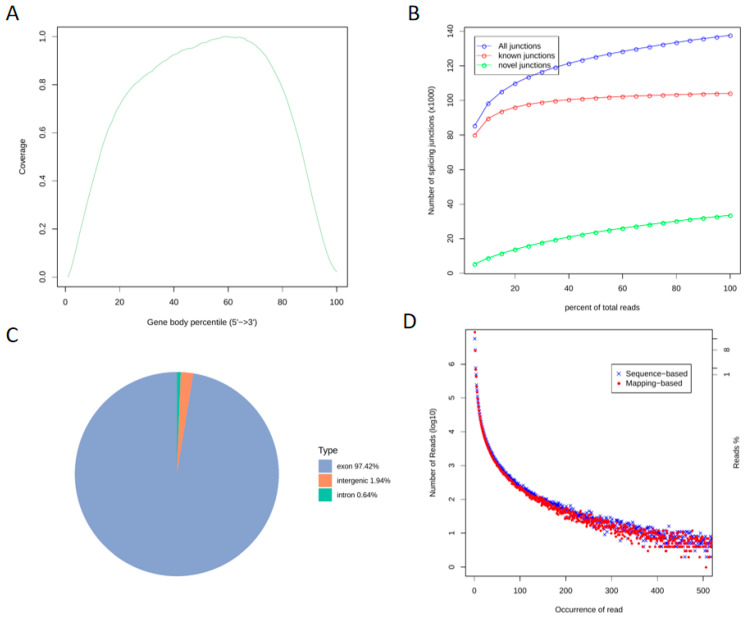
Genomic Regional Distribution and Transcriptome-Wide Quality Assessment ((**A**). Gene Coverage Analysis; (**B**). Gene Expression Saturation Curve; (**C**). Genomic Regional Distribution of Reads; (**D**). Duplicate Sequence Frequency Distribution).

Following transcriptome sequencing of the quality-inspected libraries, we performed quality control on the raw data to ensure the accuracy of downstream analyses (Figure 2). The gene coverage analysis comprehensively reflected the sequence coverage across the 5′ to 3′ regions of all genes in the samples, revealing no significant bias (Figure 2A), which indicates good uniformity of the sequencing experiment. Among all 140,000 splice junctions identified, over 100,000 were previously known, while more than 30,000 were novel. As the number of sequenced reads increased, the junction count also rose and stabilized, confirming that the sequencing depth met the analytical standards (Figure 2B). Analysis of the genomic regional distribution of aligned reads showed that the highest proportion (97.42%) mapped to exonic regions, followed by intergenic regions (1.94%), with the smallest fraction (0.64%) aligning to intronic regions (Figure 2C). The experimental sequencing reads largely overlapped with the reference genome-mapped reads, demonstrating robust alignment (Figure 2D). Collectively, these results indicate that the overall transcriptome quality is satisfactory for subsequent analyses.

### 3.3. Differential Gene Expression Analysis

**Figure 3:**
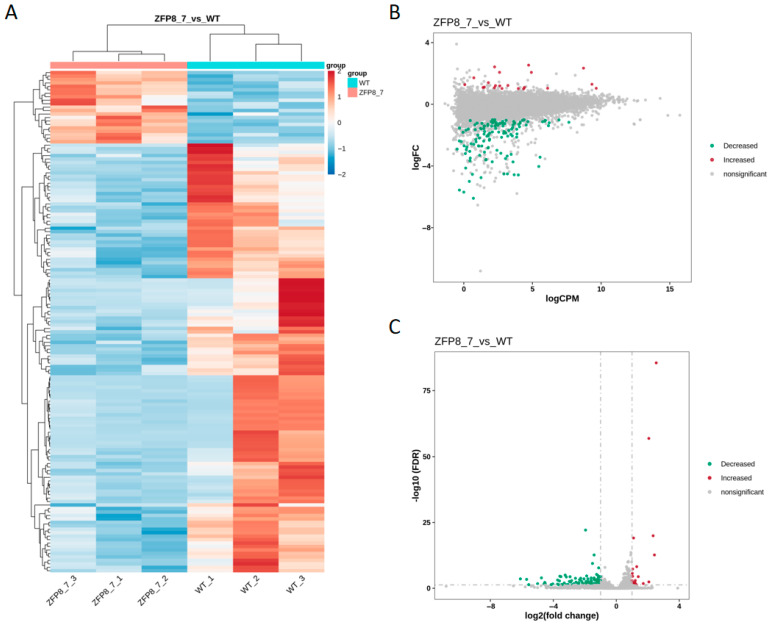
Differential Gene Expression Analysis between Transgenic Arabidopsis overexpressing *GhZFP8* from Cotton and Wild-Type Arabidopsis ((**A**). Clustering Heatmap of Differentially Expressed Genes; (**B**). M-A Plot of Differentially Expressed Genes; (**C**). Volcano Plot of Differentially Expressed Genes. In (**A**), blue represents low gene expression in the samples, while red represents high expression. In (**B**,**C**), red indicates significantly upregulated differentially expressed genes, green indicates significantly downregulated differentially expressed genes, and gray indicates genes without significant differential expression; WT_1 to WT_3 as well as ZFP8_7_1 to ZFP8_7_3 are biological repeats.).

Based on the gene expression differences in counts per million (CPM) between the *GhZFP8* transgenic group and the wild-type group, it was identified via heatmap analysis that most of the differentially expressed genes were highly expressed in the wild-type group (Figure 3A). Differentially expressed genes were screened using the criteria of |log2(fold change)| >1 and false discovery rate (FDR) <0.05. The results showed that the number of downregulated genes significantly exceeded that of upregulated genes. The distribution of CPM values and FDR values for the differentially expressed genes are presented in Figure 3B and Figure 3C, respectively.

### 3.4. GO Enrichment Analysis of Differentially Expressed Genes

**Figure 4:**
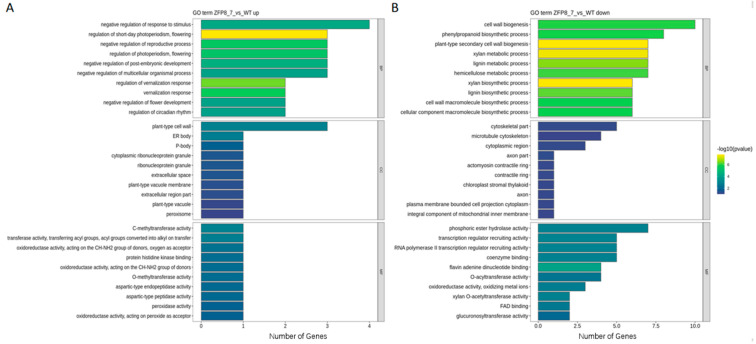
GO Enrichment Analysis of Differentially Expressed Genes ((**A**). GO Enrichment of Upregulated Genes; (**B**). GO Enrichment of Downregulated Genes. Biological Process (BP), Cellular Component (CC), and Molecular Function (MF)).

A gene enrichment approach was employed to perform functional enrichment analysis on the differentially expressed genes identified above, with the aim of identifying their potential involvement in important biological pathways. The GO enrichment analysis annotated the differentially expressed genes from three aspects: Biological Process (BP), Cellular Component (CC), and Molecular Function (MF) (Figure 4).

The results showed that, among the upregulated genes, the number of differentially expressed genes involved in biological processes was the highest, primarily concentrated in pathways such as negative regulation of stimulus response, short-day photoperiod regulation, negative regulation of reproductive processes, and photoperiod regulation. For cellular components, the differentially expressed genes were mainly enriched in plant-type cell walls and ER body composition. In terms of molecular functions, the number of annotated differentially expressed genes was the smallest, including activities such as C-methyltransferase activity, O-methyltransferase activity, and histidine kinase binding (Figure 4A). Among the downregulated genes, the enriched biological pathways were more diverse. For example, the most significant pathway enriched in biological processes was cell wall biosynthesis and phenylpropanoid biosynthesis processes. In molecular functions, the most notable enrichments were phosphatase activity and recruitment of transcriptional regulators. In cellular components, the number of enriched pathways was the smallest, including certain cytoskeletal components and microtubule cytoskeleton (Figure 4B).

### 3.5. KEGG Enrichment Analysis of Differentially Expressed Genes

**Figure 5:**
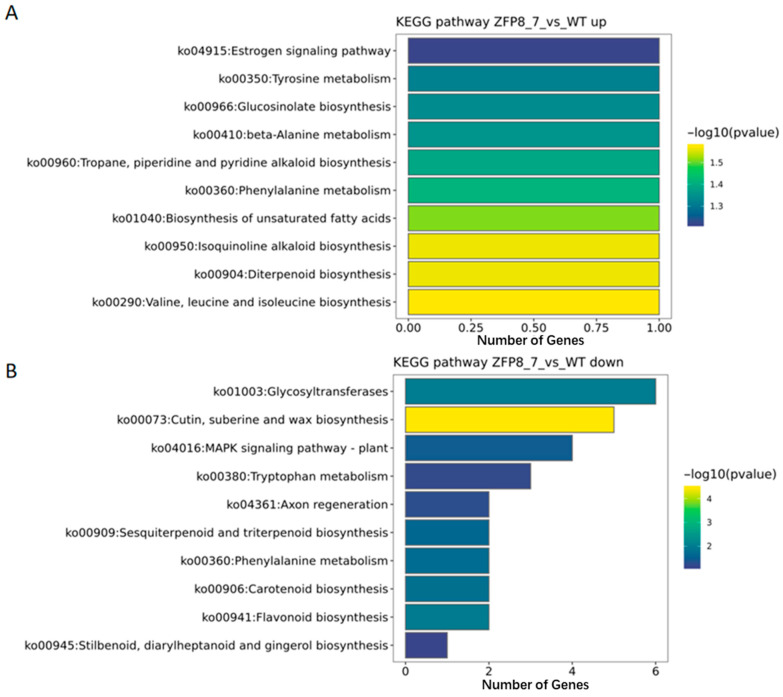
KEGG Enrichment Analysis of Differentially Expressed Genes ((**A**). KEGG Enrichment Analysis of Upregulated Genes; (**B**). KEGG Enrichment Analysis of Downregulated Genes.).

To further investigate the metabolic pathways associated with the differentially expressed genes in *GhZFP8* transgenic Arabidopsis compared to wild-type Arabidopsis, KEGG enrichment analysis was performed on both upregulated and downregulated genes (Figure 5).

Among the upregulated genes, the number of differentially expressed genes annotated to specific pathways was limited, primarily focusing on signaling pathways, biological metabolism, and biosynthesis pathways, such as the estrogen signaling pathway (ko04915: Estrogen signaling pathway), tyrosine metabolism (ko00350: Tyrosine metabolism), and glucosinolate biosynthesis (ko00966: Glucosinolate biosynthesis) (Figure 5A). In contrast, the downregulated genes were enriched in a wide range of pathways, including glycosyltransferases (ko01003: Glycosyltransferase) and the MAPK signaling pathway in plants (ko04016: MAPK signaling pathway-plant) (Figure 5B).

## 4. Discussion

Zinc finger proteins (ZFPs) have been identified in various crops, including cotton, Arabidopsis, and rice, and are known to possess diverse cellular biological functions, such as DNA and RNA binding, signal transduction, and participation in transcriptional regulation. The *GhZFP8* gene is a C2H2-type zinc finger protein gene in cotton. Current research on *GhZFP8* primarily focuses on its structural and functional analysis, expression patterns, and the generation of transgenic plants.

Plant trichomes are specialized structures developed from epidermal cells, and their growth and development are regulated by processes such as the cell cycle, transcription, and cytoskeletal functions [32,33]. With advancements in molecular biology and sequencing technologies, the identification of key genes involved in regulating trichome development has become increasingly sophisticated, yielding significant results. For instance, overexpression of the GL1 gene in wild-type Arabidopsis resulted in a noticeable reduction in trichome numbers compared to control rosette leaves [34]. In this study, we observed that compared to wild-type Arabidopsis, *GhZFP8*-transgenic Arabidopsis exhibited an increase in trichome numbers on siliques, along with inhibited growth and development.

To gain a deeper understanding of the functional role of *GhZFP8*, we conducted transcriptome analysis using RNA-seq technology to compare *GhZFP8*-transgenic Arabidopsis with wild-type Arabidopsis. In the differential expression analysis, we found that the number of downregulated differentially expressed genes was higher than the number of upregulated genes, with most genes showing higher expression in the wild-type compared to the *GhZFP8*-transgenic group. Based on this observation, we hypothesized that the overexpression of *GhZFP8* might suppress the expression of certain genes.

In the GO enrichment analysis, the number of differentially expressed genes enriched in biological processes was the highest, with significant enrichment in pathways such as negative regulation of stimulus response, short-day photoperiod regulation, cell wall biosynthesis, and phenylpropanoid biosynthesis. In terms of cellular components, the number of enriched genes was the lowest, primarily concentrated in plant-type cell walls and cytoskeletal components. For molecular functions, the enriched genes were mainly associated with activities such as C-methyltransferase activity and phosphatase activity.

In the KEGG enrichment analysis, the number of upregulated genes annotated to specific pathways was limited, primarily focusing on pathways such as the estrogen signaling pathway, tyrosine metabolism, and glucosinolate biosynthesis. In contrast, the downregulated genes were enriched in a wide range of pathways, such as glycosyltransferases and the MAPK signaling pathway in plants. Previous studies have shown that plant hormones, such as gibberellins (GA), cytokinins (CK), and jasmonic acid (JA), can regulate trichome development [35]. Through this study, we have provided valuable information for further elucidating the functional role of the *GhZFP8* gene in cotton and its potential application in cotton genetic improvement.

## 5. Important Implication and Future Perspectives of This Study

This study reveals the regulatory role of cotton’s *GhZFP8* gene in trichome development and plant growth—by inducing more trichomes and inhibiting growth when overexpressed in Arabidopsis, it provides direct evidence that *GhZFP8* is a candidate gene involved in shaping epidermal traits and developmental processes of plants. Second, the transcriptome findings (e.g., more downregulated differentially expressed genes, enrichment in cell wall biosynthesis, phenylpropanoid biosynthesis, and MAPK signaling pathway) link *GhZFP8* to core metabolic and signaling networks, laying a foundation for understanding the molecular mechanism underlying its functional role in cotton. Additionally, these results highlight *GhZFP8*’s potential value for cotton genetic improvement, as trichome-related traits are closely associated with cotton fiber quality and stress resistance.

In future research we aim to: further verify the direct target genes of *GhZFP8* (e.g., genes in the MAPK pathway or cell wall biosynthesis) through molecular experiments (e.g., ChIP-seq, yeast one-hybrid) to clarify how it regulates downstream pathways; second, conduct functional validation of *GhZFP8* in cotton itself (rather than *Arabidopsis*) to confirm its role in cotton trichome/fiber development and growth, avoiding limitations of heterologous expression systems; third, explore the application of *GhZFP8* in cotton breeding—for example, by modulating its expression to optimize fiber yield/quality or enhance stress resistance, and combining it with other trichome-regulating genes (e.g., GL1 homologs in cotton) to develop more efficient genetic improvement strategies.

## 6. Conclusions

Transcriptional profiling revealed that a significant proportion of differentially expressed genes were downregulated, while fewer were upregulated. Among the upregulated genes, the majority were involved in biological processes, with the highest number of differentially expressed genes concentrated in pathways such as negative regulation of stimulus response, short-day photoperiod regulation, negative regulation of reproductive processes, and photoperiod regulation. Genes annotated to cellular components were primarily enriched in plant-type cell walls and ER body composition, while those annotated to molecular functions were the least represented. For the downregulated genes, the enriched biological pathways were more diverse, with the most significant pathways being cell wall biosynthesis and phenylpropanoid biosynthesis. In terms of cellular components, the number of enriched pathways was the smallest.

In metabolic pathways, the upregulated genes annotated to specific pathways were limited, primarily focusing on signaling pathways, biological metabolism, and biosynthesis pathways. In contrast, the downregulated genes were enriched in a wider range of pathways, such as glycosyltransferases.

## Figures and Tables

**Table 1:** PCR Amplification.

Temperature	TIME	CYCLE
98 °C	30 s	--
98 °C	10 s	18 Cycles
65 °C	30 s	18 Cycles
72 °C	45 s	18 Cycles
72 °C	5 min	--
4 °C	∞	--

## Data Availability

The original contributions presented in this study are included in the article. Further inquiries can be directed to the corresponding author.
